# Recent Advances in Metal Oxide Electron Transport Layers for Enhancing the Performance of Perovskite Solar Cells

**DOI:** 10.3390/ma17112722

**Published:** 2024-06-03

**Authors:** Ying-Han Liao, Yin-Hsuan Chang, Ting-Han Lin, Kun-Mu Lee, Ming-Chung Wu

**Affiliations:** 1 Department of Chemical and Materials Engineering, Chang Gung University, Taoyuan 333323, Taiwan; yinghanliao@cgu.edu.tw (Y.-H.L.); yinhsuanchang@cgu.edu.tw (Y.-H.C.); tinghanlin@cgu.edu.tw (T.-H.L.); kmlee@cgu.edu.tw (K.-M.L.); 2 Center for Sustainability and Energy Technologies, Chang Gung University, Taoyuan 333423, Taiwan; 3 Department of Materials Engineering, Ming-Chi University of Technology, New Taipei City 243303, Taiwan; 4 Division of Neonatology, Department of Pediatrics, Chang Gung Memorial Hospital at Linkou, Taoyuan 333423, Taiwan

**Keywords:** metal-doped TiO_2_, planar electron transport layer, mesoporous electron transport layer, SnO_2_, passivation, perovskite solar cell

## Abstract

Perovskite solar cells (PSCs) have attracted considerable interest owing to their low processing costs and high efficiency. A crucial component of these devices is the electron transport layer (ETL), which plays a key role in extracting and transmitting light-induced electrons, modifying interfaces, and adjusting surface energy levels. This minimizes charge recombination in PSCs, a critical factor in their performance. Among the various ETL materials, titanium dioxide (TiO_2_) and tin dioxide (SnO_2_) stand out due to their excellent electron mobility, suitable band alignment, high transparency, and stability. TiO_2_ is widely used because of its appropriate conduction band position, easy fabrication, and favorable charge extraction properties. SnO_2_, on the other hand, offers higher electron mobility, better stability under UV illumination, and lower processing temperatures, making it a promising alternative. This paper summarizes the latest advancements in the research of electron transport materials, including material selection and a discussion of electron collection. Additionally, it examines doping techniques that enhance electron mobility and surface modification technologies that improve interface quality and reduce recombination. The impact of these parameters on the performance and passivation behavior of PSCs is also examined. Technological advancements in the ETL, especially those involving TiO_2_ and SnO_2_, are currently a prominent research direction for achieving high-efficiency PSCs. This review covers the current state and future directions in ETL research for PSCs, highlighting the crucial role of TiO_2_ and SnO_2_ in enhancing device performance.

## 1. Introduction

The energy shortage has become a pressing global concern, intensifying the urgency for a transition to sustainable energy sources. This situation has especially motivated scientists worldwide to concentrate their efforts on enhancing the power conversion efficiency (PCE) of solar energy technologies and accelerating their advancement. It is estimated that by 2050, sun-derived power will become the predominant energy source worldwide [1]. Organic-inorganic halide perovskite solar cells (PSCs) have drawn extraordinary consideration for being the most commercially feasible solar cells. Their remarkable attributes, including outstanding photoelectric properties, solution-based processes, long electron-hole diffusion lengths, low cost, and outstanding photovoltaic performance, have captured the imagination of researchers and industry. Over recent years, PSCs have achieved an astounding leap in their highest certified PCE, surging from 3.8% to an impressive 26.1% [2,3].

**Figure 1:**
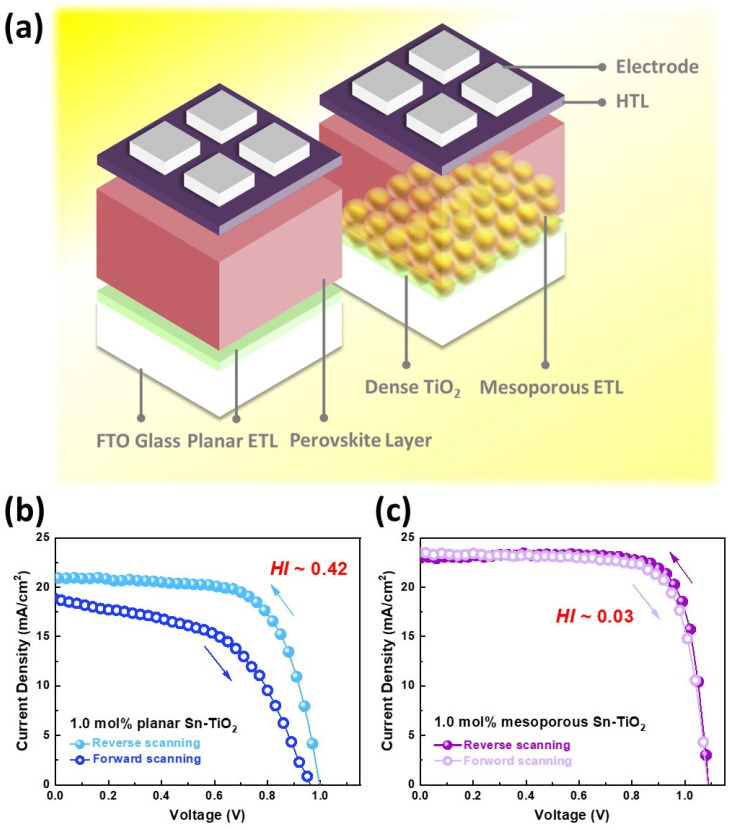
(**a**) The device structure of planar and mesoporous n-i-p structured perovskite solar cells and their J-V curve hysteresis behaviors in the (**b**) planar and (**c**) mesoporous n-i-p structured perovskite solar cells [17,18].

In the initial stages of the PSCs era, researchers drew inspiration from concepts and materials employed in dye-sensitized solar cells (DSSCs) and organic photovoltaics (OPV) [4,5]. The relationship among the electron transport layer (ETL), the perovskite active layer, and the hole transport layer (HTL) has been well established and is known to impact the performance of PSCs significantly. In early research, planar ETLs were extensively utilized in PSCs and were found to exhibit high hysteresis effects. This problem is attributed to the interaction of migrating ions (e.g., I^−^, Br^−^, or MA^+^) with the TiO_2_ layer. The low surface area increased the carrier flux imbalance and interfacial charge accumulation between the TiO_2_ ETL and the perovskite layer [6,7]. The search for more stable, adaptable, and efficient PSCs led to the investigation of modification of ETL. Therefore, researchers developed a new ETL structure, mesoporous TiO_2_, to replace the traditional planar ETL. Figure 1a shows the device structure of planar and mesoporous n-i-p structured PSCs. Gratzel et al. pioneered the use of a mesoporous TiO_2_ structure as the ETL, demonstrating efficient PCE [8]. This approach enabled the integration of the perovskite active layer into the mesoporous structure. Moreover, the mesoporous TiO_2_ ETL enlarges the effective interface, improving charge transfer, especially when the diffusion lengths of holes and electrons are either short or imbalanced [9]. Some literature also mentions that meso-TiO_2_ scaffolds have been reported to act as barriers to moisture. The hydroxyl group or physisorbed water molecules on TiO_2_ surfaces could avoid the formation of unexpected PbI_2_ crystals. Thus, it protects the active layer and contributes to the stability of the device’s performance over time [10,11,12,13].

Nevertheless, issues concerning electron mobility and shallow and deep defect levels in TiO_2_ persisted, driving the exploration of straightforward and practical strategies, such as metal-ion doping, to further enhance its performance.

Hysteresis in PSCs refers to the phenomenon where the current-voltage (J-V) characteristics of the cell exhibit a dependence on the direction and speed of the voltage sweep during measurement. This results in different J-V curves when the voltage is scanned from a lower to a higher value (forward scan) compared to when it is scanned from a higher to a lower value (reverse scan). The presence of hysteresis in perovskite solar cells can affect the reproducibility and stability of the measured photovoltaic parameters, such as the open-circuit voltage (V*_OC_*), short-circuit current (J*_SC_*), fill factor (FF), and overall efficiency. V*oc* is the maximum voltage a solar cell can produce when no current is flowing. It measures the electric potential difference between the cell’s terminals under illumination and is influenced by the semiconductor materials, the quality of the junction, the perovskite material’s bandgap, the built-in potential, and charge carrier recombination rates. J*_SC_* is the maximum current that flows through a solar cell when its terminals are shorted, meaning the voltage across the cell is zero. It represents the current generated under standard illumination conditions and depends on the light absorption properties, charge separation and collection efficiency, and the quality of contacts and interfaces. Higher J*_SC_* values indicate better light-to-electric current conversion. FF describes the shape of the current-voltage (J-V) curve and is the ratio of the maximum power output (Pm) to the product of V*_OC_* and J*_SC_*. It measures the cell’s quality, which is influenced by series and shunt resistances, material quality, and interfaces. A higher FF indicates a more efficient solar cell with lower resistive losses and better charge carrier extraction, crucial for maximizing power conversion efficiency in perovskite solar cells.

The exact mechanisms behind hysteresis are complex and not fully understood, but several factors have been identified as potential contributors: (1) Ion Migration: mobile ions within the perovskite material can redistribute in response to electric fields, altering the internal electric field and charge distribution. (2) Interfacial Charge Accumulation: charges can accumulate at interfaces between layers in the solar cell, such as between the perovskite layer and the charge transport layers, leading to imbalanced charge transport and recombination dynamics. (3) Capacitive Effects: the dielectric properties of materials and interfaces within the cell can contribute to capacitive effects that influence charge dynamics during the voltage sweep.

Researchers aim to minimize hysteresis in perovskite solar cells to improve their performance and reliability, exploring various material and structural modifications to achieve more stable and reproducible behavior. Overcoming the hysteresis phenomenon in n-i-p structured devices during the measurement of photovoltaic conversion efficiency poses a significant challenge. This phenomenon complicates the estimation of the actual operational photovoltaic conversion efficiency of these devices. Enhancing the carrier mobility of the transport layer, particularly in the poorly conductive TiO_2_ layer, and reducing the defect density can improve carrier collection and further reduce the hysteresis effect in perovskite solar cells during measurements. To quantify and understand the hysteresis phenomenon, many research groups evaluate the hysteresis index (HI) of solar cells, calculated as follows:

(1) $$HI=\frac{J_{RS}\left(0.8V_{OC}\right)-J_{FS}(0.8V_{OC})}{J_{RS}(0.8V_{OC})}$$

*J_RS_*(0.8*V_OC_*) and *J_F__S_*(0.8*V_OC_*) represent the photocurrent densities at a bias of 80% *V_OC_* during reverse and forward bias photovoltaic measurements, respectively. The utility of the hysteresis index lies in its ability to quantify the extent of performance variation due to charge trapping, ion migration, and interfacial phenomena within the PSCs. A higher hysteresis index indicates a greater disparity between the forward and reverse scan results, suggesting more pronounced charge recombination or ion migration issues. Conversely, a lower hysteresis index denotes more stable and consistent performance, implying that the device is less affected by these transient effects [14,15,16]. Figure 1b,c are the J-V curve hysteresis behaviors in the planar and mesoporous perovskite solar cells demonstrated by Wu et al. [17,18]. In order to make a more complete comparison, we further conduct hysteresis analysis based on our planar Sn-TiO_2_ structure, and the HI value is 0.42. Figure 1c shows that perovskite solar cells with metal-doped mesoporous TiO_2_ as ETL exhibit minor hysteresis. Comparing the forward and reverse scan J-V curves of planar and mesoporous TiO_2_, the current difference between forward and reverse scans is minimal, with an HI value of only 0.03. The mesoporous structure provides a larger surface area for better interface contact, improves charge transport, better manages ion migration, and reduces recombination. These factors contribute to a more stable and efficient charge extraction process, which minimizes the transient effects that lead to hysteresis.

Recent research has explored SnO_2_ as a promising ETL for PSC due to its wide optical bandgap (3.6–4.0 eV), high electron mobility, and favorable band alignment at the ETL/perovskite interface [19,20,21]. Considering these developments, several straightforward coating techniques have been employed to achieve high-quality ETL for perovskite solar cells. These methods include the deposition of SnO_2_ nanoparticles, thermal oxidation of Sn (IV) isopropoxide, chemical bath deposition, and atomic layer deposition [22,23,24]. In addition to the deposition methods, considering the non-radiative recombination of SnO_2_-based PSCs, extensive research has focused on modifying SnO_2_ ETL through surface passivation, controlling the energy level, and increasing charge transport by doping [25].

Throughout the development of PSCs, thanks to numerous research projects, innovations in material synthesis, device design, and fabrication methods have improved understanding of the critical role of the ETL/perovskite active layer in enhancing their performance. While PSCs have witnessed significant advancements, there are still numerous challenges and barriers, both fundamental and practical, that need to be overcome to establish their commercial feasibility. This review will delve into the recent advancements in ETL materials for PSCs. In the following paragraphs of this article, we first summarize the progress of TiO_2_-based ETL. Then, we discuss the design of SnO_2_-based ETL. The last segment will examine the interfacial passivation strategies for the SnO_2_/perovskite active layer. In light of this background, the ETL/perovskite active layer is positioned as a beacon of hope in the search for effective and long-lasting solar energy conversion technologies. This background reveals an enthralling journey of exploration, innovation, and knowledge dissemination. In summary, we provide a concise overview and brief insights on advancing PSCs toward efficient and stable solar-to-power technology and offer predictions for their future.

## 2. The Advantages and Disadvantages of Perovskite Solar Cells

**Figure 2:**
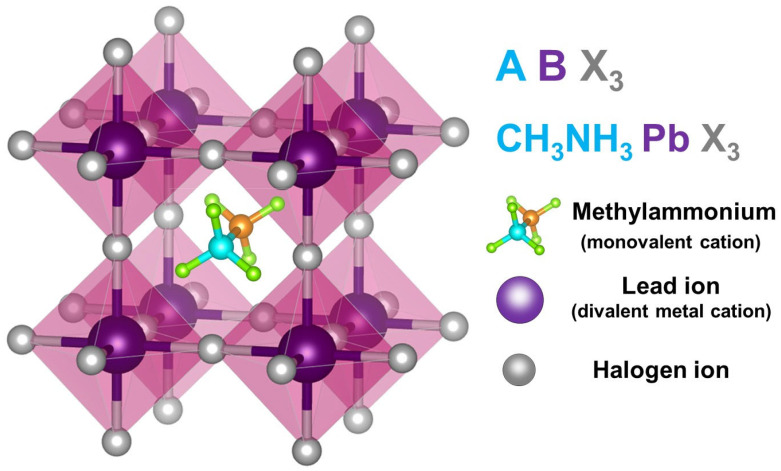
Crystal structure of organic-inorganic lead halide-based material with the generic chemical formula ABX_3_.

Perovskite solar cells are a type of solar cell that utilizes perovskite-structured materials in their light-harvesting layer. These devices comprise a crystal structure made from organic-inorganic lead halide-based materials, often referred to as ABX_3,_ as shown in Figure 2. In this structure, ‘A’ represents a monovalent cation, ‘B’ is a bivalent metal cation, and ‘X’ is a halogen anion. The stability of these devices comes from an octahedral structure formed when six halogen ions surround a lead ion [26]. These octahedrons are interconnected, with a monovalent cation, typically methylammonium, positioned at the center of each one.

**Figure 3:**
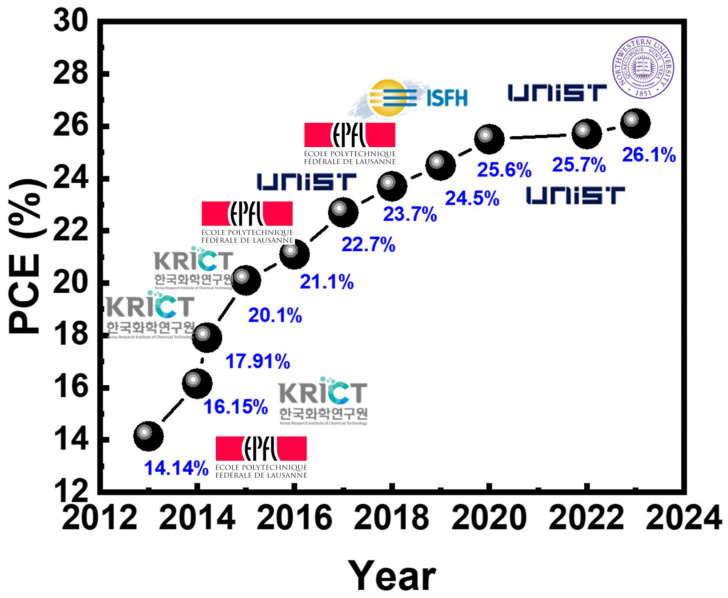
Trends in high-performing perovskite solar cells according to the National Renewable Energy Laboratory’s record of best research-cell efficiencies.

Prof. Miyasaka reported a perovskite solar cell in a 2009 JACS article, demonstrating a power conversion efficiency (PCE) of about 3.8% [3]. Figure 3 shows the trends in high-performing PSCs according to the National Renewable Energy Laboratory’s (NREL) record of best research-cell efficiencies. Since then, according to the NREL’s record of best research-cell efficiencies, the PCE of these devices has rapidly increased to 26.1% [2,27,28]. The devices reported by Sargent et al. employ a p–i–n stack of FTO/SAMs/perovskite/C60/SnOx/Ag. This marked increase in efficiency underscores the potential of PSCs in renewable energy. Perovskite solar cells offer several advantages, including high efficiency, a tunable bandgap, low production costs, solution processing, and flexibility. One of the primary limitations of these materials is their vulnerability to moisture, which leads to accelerated degradation and diminished PCE under humid conditions. Concerns regarding certain perovskite constituents’ environmental and health implications, especially lead, also pose significant challenges. Additionally, the performance of the PSC is affected by hysteresis, and there is a restricted capacity to adjust their bandgap, further complicating their application.

The hysteresis effect observed during solar cell measurements refers to the incomplete overlap of the current density-voltage (J-V) curves when sweeping the voltage from negative to positive (forward scan) and from positive to negative (reverse scan). The hysteresis not only impacts the precision of measurements but also influences the overall performance of PSCs. PSCs are typically categorized into two configurations: n-i-p and p-i-n structures. Given the inherent p-type semiconductor nature of perovskite materials, n-i-p structured PSCs, with their shorter electron transport paths, are more likely to achieve high photovoltaic performance and are thus widely utilized. However, a significant challenge in n-i-p structured PSCs is the hysteresis effect observed during measurements [29,30]. Contributing factors to this hysteresis include (1) lower electron mobility in the titanium dioxide (TiO_2_) ETL, affecting the balance of electron and hole carrier fluxes [31]; (2) shallow trap states near the conduction band formed by oxygen vacancies, cation interstitials, and cation Frenkel defects, leading to charge recombination [32]; (3) the pathways and contact interfaces between the ETL and the perovskite active layer, playing a crucial role in charge transport [33].

## 3. Metal-Doped TiO_2_ Electron Transport Layer for Perovskite Solar Cells

TiO_2_ is widely used in the ETL of PSCs due to its low cost, non-toxicity, chemical and optical stability, and suitable conduction band alignment. Enhancing charge collection, conductivity, charge transport efficiency, and reducing electron-hole recombination can be efficiently achieved by doping TiO_2_ with new ions. Alkali metal doping, such as with Li, Na, and K, improves electron conductivity and surface defect passivation, with Li-doping notably reducing surface defects and Cs-doping enhancing pore-filling and uniformity of the perovskite layer. Alkali-earth metals like Mg and Sr further enhance device performance; Mg-doped TiO_2_ achieves a better energy match with the absorber, leading to higher V*_OC_* and FF. Additionally, p-block elements (e.g., B, Al, In, C, Sn, Pb, Cl) boost electron collection and transfer properties, with B-TiO_2_ and Sn-TiO_2_ effectively reducing hysteresis. Incorporating d-block elements, such as Zr, in TiO_2_ significantly enhances conduction, suppresses electron trap-states, and minimizes leakage paths, thereby improving PCE and overall device performance [34,35,36,37]. Various metals, such as Cs, Mg, Ag, Zn, and Sn, have been extensively studied for doping [18,38,39,40,41] (Table 1). Cs-TiO_2_, synthesized by the sol-gel method, has been integrated into planar-structure PSCs. The Cs doping causes an upshift in the Fermi level of the TiO_2_ compact layer, resulting in an increase in the V*_OC_* of the PSCs without reducing the J*_SC_*, due to the enhanced conductivity of the compact layer [38]. Mg-doped TiO_2_ ETLs exhibit improved optical transmission properties, an upshifted conduction band minimum (CBM), and a downshifted valence band maximum (VBM). This doping enhances the hole-blocking effect and increases electron lifetime, contributing to better overall performance in PSCs [41]. Ag-TiO_2_ ETL causes the short-circuit current density to increase notably because of the lower electron–hole recombination, which increases the electron injection. In addition, with Ag doping, the hysteresis behavior can be hindered [39]. Zn-doped TiO_2_ compact layers prepared by the sol-gel method can tune the optical absorption behavior, electrical conductivity, surface morphology, and charge carrier dynamics. A Zn-doped TiO_2_ compact layer exhibits high charge separation rate, which could be attributed to the high charge carrier transport of Zn-doped TiO_2_ [40]. When Sn is doped into TiO_2_, it facilitates the formation of an anatase/rutile phase junction at a fixed calcination temperature of 550 °C. Compared to pristine TiO_2_ with a pure anatase phase, mixed-phase TiO_2_ exhibits a reduced bandgap and higher charge carrier mobility [18]. The appropriate dopant can adjust the optical characteristics, improve the separation of electron-hole pairs, and optimize the band alignment between the ETL and the perovskite active layer, enhancing charge transportation within the device. However, the poor contact between a planar ETL and the perovskite layer and the low conductivity of pristine planar TiO_2_ ETL contribute to the hysteresis phenomenon in perovskite solar cells.

The mesoporous TiO_2_ structure, providing intimate contact with the perovskite layer, allows the perovskite to infiltrate into its mesoporous microstructure and offers a large contact area for electron transport. This close contact also reduces the defect density at the interface. The mesoporous microstructure facilitates the penetration of the perovskite active layer, significantly increasing the contact area and shortening the electron transport path [8,9,42,43,44,45,46]. This compensates for the poor electron mobility of TiO_2_ and improves the balance in electron and hole transport fluxes. Additionally, the close contact between the mesoporous TiO_2_ electron transport layer and the perovskite layer effectively reduces the density of interface defects. In summary, increasing the contact area between the electron transport layer and the perovskite layer, reducing interface defect density, and enhancing the electron transport capability of TiO_2_ can effectively mitigate the hysteresis effect.

As mentioned earlier, the development of metal-doped mesoporous TiO_2_ for use in ETL holds great promise for enhancing electron properties and passivating defects, as detailed in Table 2. Employing metal-doped mesoporous TiO_2_ is particularly effective in augmenting the ETL’s electron conductivity and reducing interface defects, which can further tune the offset of the band gap of pure TiO_2_. These improvements are crucial for enhancing the electron transport characteristics in perovskite solar cells and mitigating the hysteresis phenomenon observed in these devices. With the doping of W into TiO_2_, the conductivity can be enhanced. The shift of the conduction band can also boost the extraction of electrons [47]. Y^3+^-substituted TiO_2_ demonstrates higher absorbance than pure TiO_2_ from the visible to the near-infrared (IR) region, indicating that more CH_3_NH_3_PbI_3_ is supported by Y–TiO_2_. Due to the poor solubility of yttrium in TiO_2_, Y_2_O_3_ segregates at the surface, facilitating increased material loading and enhancing the light absorption of the deposited perovskite on TiO_2_ [48]. Doping TiO_2_ with aluminum (Al) enhances electron mobility, leading to an increase in FF. This improvement in electron mobility, attributed to the enhanced conductivity, results in a PCE of 14.1% [49]. With the doping of Ce in TiO_2_ precursor, the PCE was boosted to 17.75% due to the optimized morphology of mesoporous TiO_2_, which enhances the electron extraction ability [50]. Doping meso-TiO_2_ with rare-earth europium ions (Eu^3+^) upshifts the Fermi level by scavenging oxygen atoms and introducing oxygen vacancies on the surface. This leads to lower series resistance and faster charge transport in the ETL, significantly enhancing performance [51]. The exceptional band alignment between the active layer and meso-Zn:TiO_2_ ETL suppresses electron-hole recombination and improves electron transfer behavior. Doping Zn ions into TiO_2_ can increase conductivity and mobility, reduce trap density, and further eliminate J–V hysteresis [52].

A significant aspect of this advancement is the role of Sn doping in TiO_2_. The energy diagram of Sn has a relatively shallow distribution; therefore, when doped into TiO_2_, Sn causes a slight upward shift in the conduction band minimum and the valence band maximum. This shift is beneficial as it leads to a higher open-circuit voltage (V*_OC_*), a key parameter in solar cell efficiency. Furthermore, the optimal band alignment between the ETL and the perovskite solar cell active layer, achieved with Sn doping, facilitates the efficient injection of electrons into the ETL. This enhancement not only improves overall device performance but also significantly reduces defect states, further decreasing charge recombination and accumulation. Overall, incorporating Sn-doped TiO_2_ into the mesoporous structure of the ETL represents a noteworthy advancement in the field of perovskite solar cell technology, offering a pathway to more efficient and stable solar cells.

## 4. Surface Modification of SnO_2_ Electron Transport Layer for Perovskite Solar Cells

Although TiO_2_ ETL exhibits outstanding performance in PSCs, there are still some limitations to its commercialization. TiO_2_ requires high-temperature processing, which is incompatible with flexible substrates and cost-effective manufacturing. Additionally, TiO_2_ has a high density of trap states that can act as recombination centers, thereby reducing overall device efficiency. Its relatively low electron mobility further limits charge transfer efficiency, and its photocatalytic activity under UV light can degrade the perovskite layer and other organic components, compromising long-term stability. In contrast, SnO_2_ can be processed at low temperatures, making it compatible with cost-effective roll-to-roll manufacturing processes and cost-effective manufacturing. Its better energy level alignment increases the open-circuit voltage. Furthermore, SnO_2_’s high transparency allows the perovskite layer to maximize light absorption, thereby increasing photocurrent and overall device efficiency. These properties make SnO_2_ an excellent alternative to TiO_2_, promising more efficient, stable, and versatile perovskite solar cells.

In recent research, substituting planar or mesoporous TiO_2_ structures with alternative inorganic metal oxides has garnered significant attention. Within planar heterojunction PSCs, tin oxide (SnO_2_) has emerged as a suitable alternative to TiO_2_ due to its higher electrical conductivity and an appropriate Fermi level relative to perovskites. The SnO_2_ layer can be quickly produced through a spin-coating process using commercially available colloidal dispersions of nanocrystalline SnO_2_. This process yields a thin, dense layer of stacked SnO_2_ nanoparticles suitable for various device architectures. Additionally, SnO_2_ demonstrates a band structure synchronized with the perovskite absorber and enhanced electron mobility, effectively mitigating interfacial carrier accumulation and the resulting current-voltage (J-V) hysteresis in solar cells.

**Figure 4:**
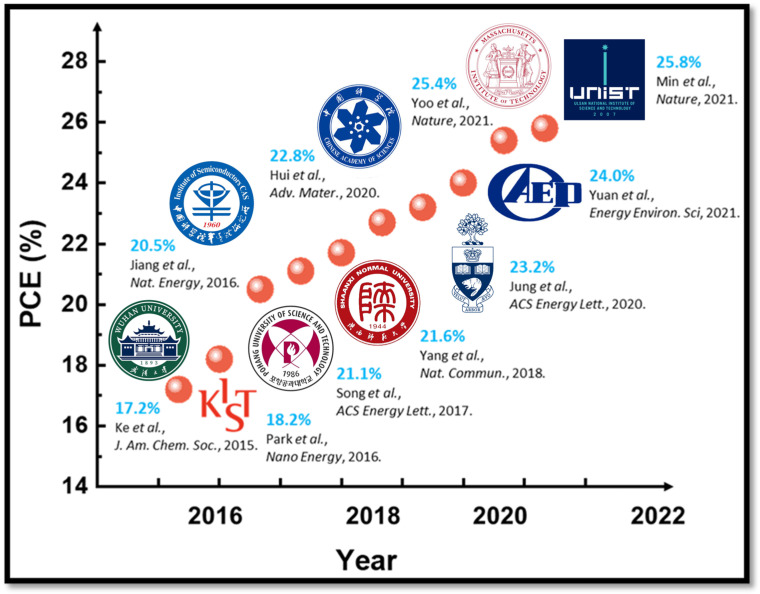
Trends in photovoltaic performance of PSCs based on SnO_2_ ETL [56,57,58,59,60,61,62,63,64,65].

We have summarized the significant advancements in applying SnO_2_ in PSCs from 2015, covering various innovative preparation methods and performance enhancement strategies in Table 3. Figure 4 shows the trends in PSCs based on SnO_2_ ETL since 2015. Starting in 2015, Ke et al. first reported using a low-temperature solution process to prepare SnO_2_ as the ETL for planar heterojunction PSCs, marking a significant progression in applying SnO_2_. The best-performing planar PSCs based on nanocrystalline SnO_2_ ETL achieved a PCE of 17.21% [56].

Subsequently, in 2016, Park et al. demonstrated a method for preparing SnO_2_ thin films through a low-temperature solution process in which the SnO_2_ is doped with lithium [57]. This modification improved the work function of transparent electrodes, increased conductivity, and facilitated electron injection and transfer. These enhancements led to improved V*_OC_*, J*_SC_*, and *FF* in PSCs. The PCE of devices with rigid and flexible architectures reached 18.2% and 14.78%, respectively. In 2016, Jiang et al. reported low-temperature solution-processed SnO_2_ nanoparticles as ETL for PSCs, demonstrating a PCE of 20.5% with minimal hysteresis [58].

In 2017, Song et al. reported a bilayer SnO_2_@a-TiO_2_ ETL on FTO for perovskite solar cells. Such a structure shows a sizeable free energy difference (ΔG) and defect-free physical contact, demonstrating a J−V scan efficiency of 21.1% and reduced hysteresis [59]. In 2018, Yang et al. reported an effective EDTA-SnO_2_ ETL that increased the PCE to 21.60%, with a certified efficiency of 21.52%. The results show that the Fermi level of EDTA-complexed SnO_2_ is much more suitable to the conduction band of perovskite, leading to high V*_OC_*. In addition, the electron mobility is also enhanced [60].

By 2020, Hui et al. significantly enhanced electron mobility by doping low-temperature solution-processed SnO_2_ with carboxylic-acid- and hydroxyl-rich red-carbon quantum dots (RCQs) as ETL. This innovation in planar PSCs increased PCE from 19.15% to 22.77% [61]. In the same year, Jung et al. performed a bifunctional surface treatment using ammonium fluoride (NH_4_F) to diminish defect sites and alter the Fermi level of SnO_2_ thin films, achieving a PCE of 23.2% [62]. Yoo et al. optimized the composition of SnO_2_ ETL, thickness, and film coverage by adjusting the parameters of chemical bath deposition. Subsequently, they decoupled the passivation strategy with methylammonium chloride (MACl) additives, which can promote perovskite crystal grain growth and achieve a certified PCE of 25.2% [64].

Yuan et al. reported efficient electron transport materials for PSCs, utilizing low-temperature solution-processed SnO_2_ nanocrystals (SnO_2_ NCs) enveloped by amorphous NbO_x_ (SnO_2_/NbO_x_). This configuration yielded a notable PCE of 24.01% with negligible hysteresis [63]. In 2021, Min et al. introduced a coherent interface between a perovskite thin film and a Cl-doped SnO_2_ electrode. This coherent interface enhanced charge extraction and transport from the perovskite layer while reducing interfacial defects. As a result, the PCE has achieved 25.8% (25.5% certified), maintaining high stability during 500 h of maximum power point tracking [65].

## 5. Dual Passivation of Perovskite and SnO_2_ ETL for Perovskite Solar Cells

Although introducing various dopants into SnO_2_ ETLs can minimize defects like vacancies, interstitials, and antisites, and cause the Fermi level of the ETL to better align with the conduction band of perovskite for improved carrier transport, there remain some unresolved challenges. Efficient PSCs crucially depend on high-quality perovskite films. Various techniques, such as encapsulation [66,67], ultraviolet filtration [68,69], replacement of MA with more stable cations [70,71], and modification, have been used to delay the degradation of perovskite materials under the stress of environmental and device operational factors for maintaining the long-term stability of PSCs. Effective encapsulation for PSCs is crucial to eliminate extrinsic instability. Encapsulation isolates the device from oxygen and moisture exposure while enhancing heat and mechanical stability, allowing it to function under varying weather conditions. UV radiation can cause photo-induced degradation in perovskite materials, leading to a rapid decrease in the efficiency of the solar cells. Besides, it can also degrade the encapsulation materials used to protect PSCs. By employing various methods such as UV-blocking coatings, absorbing layers, and reflective coatings, PSCs can be protected from the harmful effects of UV radiation. For the mix-cation perovskite, the substitution of a larger ionic radius of FA cations (2.53 Å) with MA cations (2.17 Å) resulted in a tolerance factor within the range of 0.9 to 1. This substitution could reduce defects in the crystal structure and improve its stability at room temperature. Yet, solution-processed perovskite films tend to exhibit lattice defects [72]. From this viewpoint, interfacial modification of SnO_2_/perovskite heterointerface is an effective method to suppress interface defects and improve the photovoltaic performance of PSCs. Here, we summarize the state-of-the-art dual passivation SnO_2_-based PSCs, as shown in Table 4.

Li et al. introduced a dual-passivation strategy for perovskite materials, targeting both cation and anion vacancies [73]. They achieved simultaneous passivation by incorporating fluoride ions, known for their high electronegativity, resulting in enhanced ionic bonding. This approach notably improved the efficiency of PSCs to 21.92%, maintaining an impressive 90% of initial PCE even after 1000 h under operational conditions. The passivation effects of alkali metals such as Na^+^, K^+^, and Li^+^ on perovskite defects have been thoroughly explored. Bu et al. synthesized CsFAMAPbI_x_Br_3-x_ by introducing K^+^, resulting in increased grain size and an enhanced PSC performance of approximately 20.56% [74]. On the other hand, I^–^ plays a dual role by eliminating iodide vacancies in the perovskite film, while K^+^ forms ionic interactions with uncoordinated halides along the grain boundaries and surfaces. This proves advantageous in suppressing ion migration. Many believe that introducing controllable excess PbI_2_ can result in accumulation at grain boundaries which reduces defect sites and protects perovskite crystals, thereby contributing to achieving high efficiency. Enhancing the quality of perovskite crystals can be achieved by incorporating controllable excess ammonium salts into the precursors, drawing inspiration from the positive effects of excess PbI_2_ addition.

Incorporating large ammonium salts aims to create 2D and 1D structures, which offer significant stability benefits over traditional 3D perovskite structures due to their enhanced hydrophobic properties. Lee et al. demonstrate that adding 2D phenylethylammonium lead iodide (PEA2PbI4) to the precursor solution helps form the cubic phase of FAPbI_3_ perovskite. The self-assembly of 2D perovskite occurs at grain boundaries, protecting the formamidinium perovskite from moisture and limiting ion migration [75]. Li et al. investigated an innovative approach by incorporating 2D C_6_H_18_N_2_O_2_PbI_4_ (EDBEPbI_4_) microcrystals into the precursor solution. The phase-pure 2D perovskite vertically passivated the grain boundaries of the deposited 3D perovskite film. This approach allows for accumulation at grain boundaries, effectively passivating defects, enhancing stability, and maintaining charge-carrier extraction integrity [76].

Zuo et al. found that the polymers containing the mentioned functional groups were positioned at grain boundaries to prevent moisture from penetrating the perovskite film. Poly(4-vinyl pyridine) (PVP) was incorporated into the perovskite precursor. The resulting thin PVP film could spontaneously self-assemble at grain boundaries, effectively passivating defects and limiting moisture ingress [77]. Zhao et al. introduced an innovative approach utilizing polymerization-assisted grain growth [78]. They added dimethyl itaconate monomers to the PbI_2_ precursor, complemented by a 0.01% molar ratio of azobisisobutyronitrile (AIBN) as an initiator. This combination first facilitates significant interaction between the carbonyl groups of the monomers and the PbI_2_. During the subsequent annealing of PbI_2_, an in-situ polymerization occurs, leading to the attachment of bulkier polymers onto the grain boundaries through pre-existing interactions. Devices stored under a nitrogen atmosphere maintained 91.8% of their initial efficiency over 2208 h.

The inherent defects of SnO_2_, such as oxygen vacancies and tin interstitials, lead to numerous shallow trap states near the conduction band. This results in carrier recombination at the SnO_2_/perovskite interface and impedes efficient carrier transport. Blending reactive anatase titania (TAc) with SnO_2_ to form a TAc-SnO_2_ ETL improves photovoltaic performance. This enhancement is attributed to faster electron transfer, fewer traps, better charge recombination resistance, and greater stability, facilitated by TAc’s gap-filling in SnO_2_ and optimizing energy levels. TAc’s carboxyl groups also strengthen the SnO_2_/TAc bonding and reduce ion accumulation at the ETL/perovskite interface, contributing to an outstanding PCE of 20.12% [82]. Chia et al. introduced an innovative non-equilibrium, photoexcitation-induced passivation (PiP) technique utilizing ultrashort laser pulses. This ultrafast photoexcitation, combined with electron-electron and electron-phonon scattering processes, facilitates rapid electron and phonon heating, effectively enabling low-temperature annealing of SnO_2_ nanoparticle-based electron transport layers prepared via CBD. This approach achieved a certified PCE of 24.14% [79].

Additionally, Lewis acid/base organic salts and inorganic materials could passivate defects at the SnO_2_/perovskite interface and enhance the interface quality. Sha et al. employed 1-ethyl-3-methylimidazolium diethyl phosphate (EMIM DEP), an organic salt, to modify the SnO_2_ surface and passivate perovskite boundaries, significantly reducing surface/interface defect density and yielding a hysteresis-free champion PCE of over 23% [80]. Xiong et al., applying biguanide hydrochloride (BGCl) to the SnO_2_ surface through Lewis coordination/electrostatic coupling links, obtained a certified PCE of 24.4% [81].

## 6. Conclusions

The concerted efforts of numerous scholars in modifying ETL have significantly advanced the field. The highest certified PCE of PSCs has reached an impressive 26.1%. This achievement underscores the indispensable role of ETL in PSCs. This article aims to provide a comprehensive overview of metal-doped TiO_2_ ETL, the prevalent SnO_2_ layer methods, and their various passivation modification techniques. We aim to offer readers an in-depth understanding of the latest research, technologies, and achievements in this rapidly evolving domain. Despite the swift progress, the field faces numerous challenges that need addressing. These include further enhancement of the PSCs’ efficiency and improving the long-term stability of usage. Addressing these issues requires ongoing dedication and thorough investigation. For future research directions, it is imperative to focus on further optimizing the surface properties of SnO_2_ ETLs to enhance their compatibility with the perovskite layer. This includes investigating novel doping elements and surface passivation techniques that effectively reduce interface defect densities and facilitate smoother charge transfer. Additionally, exploring scalable and cost-effective fabrication techniques for high-quality SnO_2_ films will be crucial for the commercial viability of PSCs. Addressing these challenges will not only push the PCE of PSCs to new heights but also significantly improve their operational stability, paving the way for their broader adoption in photovoltaic applications.

## Figures and Tables

**Table 1:** Photovoltaic performance of perovskite solar cells based on planar metal-doped TiO_2_ electron transport layer.

Metal-Doped ETL	Device Structure	V*_OC_* (V)	J*_SC_* (mA·cm^2^)	FF (%)	PCE (%)	Ref.
Cs-TiO_2_	FTO/Cs-TiO_2_/CH_3_NH_3_PbI_3−x_Cl_x_ /P3HT/Ag	0.64	14.40	57.1	5.3	[38]
Mg-TiO_2_	FTO/Mg-compact TiO_2_/TiO_2_ /CH_3_NH_3_PbI_3_/spiro-OMeTAD/Au	1.05	18.34	62.0	12.3	[41]
Ag-TiO_2_	FTO/Ag-TiO_2_/CH_3_NH_3_PbI_3−x_Cl_x_ /spiro-OMeTAD/Ag	1.00	20.50	68.7	14.1	[39]
Zn-TiO_2_	FTO/Ag-TiO_2_/CH_3_NH_3_PbI_3−x_Cl_x_ /spiro-OMeTAD/Au	0.91	22.30	68.8	14.0	[40]
Sn-TiO_2_	FTO/Sn-TiO_2_/CH_3_NH_3_PbI_3−x_Cl_x_ /spiro-OMeTAD/Ag	0.99	21.00	69.4	14.4	[18]

**Table 2:** Photovoltaic performance of perovskite solar cells based on mesoporous metal-doped TiO_2_ electron transport layer.

Metal-Doped ETL	Device Structure	V*_OC_* (V)	J*_SC_* (mA·cm^−2^)	*FF* (%)	PCE (%)	Ref.
Au-mesoTiO_2_	FTO/Au NPs-TiO_2_/CH_3_NH_3_PbI_3_ /spiro-OMeTAD/Ag	0.80	18.70	55.0	8.8	[53]
W-mesoTiO_2_	FTO/W-meso TiO_2_ /CH_3_NH_3_PbI_3_/Carbon	0.86	20.79	59.0	10.5	[47]
Y-mesoTiO_2_	FTO/Y-mesoTiO_2_/CH_3_NH_3_PbI_3_ /spiro-OMeTAD/Au	0.95	18.10	66.0	11.2	[48]
Nb-mesoTiO_2_	FTO/c-TiO_2_/Nb-mesoTiO_2_ /CH_3_NH_3_PbI_3_/spiro-OMeTAD/Au	0.99	18.70	72.3	13.4	[54]
Al-mesoTiO_2_	FTO/c-TiO_2_/Al-mesoTiO_2_ /CH_3_NH_3_PbI_3_/spiro-OMeTAD/Au	1.07	20.86	63.0	14.1	[49]
Ag-mesoTiO_2_	FTO/c-TiO_2_/Ag-mesoTiO_2_ /CH_3_NH_3_PbI_3_/spiro-OMeTAD/Ag	1.03	22.82	75.4	17.7	[55]
Ce-mesoTiO_2_	FTO/c-TiO_2_/Ce-mesoTiO_2_ /CH_3_NH_3_PbI_3_/spiro-OMeTAD/Ag	1.05	23.61	71.7	17.8	[50]
Eu-mesoTiO_2_	FTO/c-TiO_2_/Eu-mesoTiO_2_ /CH_3_NH_3_PbI_3_/spiro-OMeTAD/Au	1.10	22.62	72.3	17.9	[51]
Zn-mesoTiO_2_	FTO/c-TiO_2_/Zn-mesoTiO_2_ /CH_3_NH_3_PbI_3_/spiro-OMeTAD/Ag	1.05	22.70	78.7	18.7	[52]
Sn-mesoTiO_2_	FTO/c-TiO_2_/Sn-mesoTiO_2_ /CH_3_NH_3_PbI_3_/spiro-OMeTAD/Ag	1.07	23.10	79.5	19.5	[17]

**Table 3:** Photovoltaic performance of perovskite solar cells based on SnO_2_ electron transport layer.

SnO_2_ ETL	Device Structure	V*_OC_* (V)	J*_SC_* (mA·cm^−2^)	*FF* (%)	PCE (%)	Ref.
SnO_2_	FTO/SnO_2_/CH_3_NH_3_PbI_3_ /spiro-OMeTAD/Au	1.11	23.27	67.0	17.2	[56]
Li:SnO_2_	FTO/Li:SnO_2_/CH_3_NH_3_PbI_3_ /spiro-OMeTAD/Au	1.01	23.27	70.7	18.2	[57]
SnO_2_	ITO/ SnO_2_/ (FAPbI_3_)_0.97_(MAPbBr_3_)_0.03_ /spiro-OMeTAD/Au	1.09	24.88	75.7	20.5	[58]
Bilayer	FTO/SnO_2_@a-TiO_2_ /perovskite/HTM/Ag	1.20	22.90	76.4	21.1	[59]
EDTA-SnO_2_	ITO/EDTA-SnO_2_/FA_0.95_Cs_0.05_PbI_3_ /spiro-OMeTAD/Au	1.11	24.57	79.2	21.6	[60]
SnO_2_-RCQs	ITO/SnO_2_-RCOs /Cs_0_._05_FA_0_._81_MA_0.14_PbI_2.55_Br_0.45_ /spiro-OMeTAD/MoO_3_/Au	1.14	24.10	82.9	22.8	[61]
NH_4_F-SnO_2_	TCO (ITO or FTO) /NH_4_F-SnO_2_/(FAPbI_3_)_0.95_(MAPbBr_3_)_0.05_ /spiro-OMeTAD/Au	1.16	24.60	81.4	23.2	[62]
SnO_2_/NbO_x_	ITO/SnO_2_/NbO_x_/FA_1-x_MA_x_PbI_3-y_Cl_y_ /spiro-OMeTAD/Au	1.18	24.95	81.6	24.0	[63]
SnO_2_	FTO/SnO_2_/(FAPbI_3_)_1-x_(MAPBBr_3_)_x_ /spiro-OMeTAD/Au	1.19	25.09	84.7	25.4	[64]
Cl-SnO_2_	FTO/SnO_2_/FASnCl_x_/FAPbI_3_ /spiro-OMeTAD/Au	1.19	25.71	84.4	25.8	[65]

**Table 4:** Enhanced photovoltaic efficiency in PSCs using Sn-doped TiO_2_ ETL with surface passivation.

Passivator	Device Structure	V*_OC_* (V)	J*_SC_* (mA·cm^−2^)	*FF* (%)	PCE (%)	Ref.
NaF/KF	ITO/SnO_2_/CsFAMA-NaF /spiro-OMeTAD/Au	1.13	24.23	80.4	21.9	[73]
KI	FTO/SnO_2_/KCsFAMAPbI_x_Br_3-x_ /spiro-OMeTAD/Au	1.13	22.95	79.0	20.6	[74]
PEAI	ITO/SnO_2_/FA_0.98_Cs_0.02_PbI_3_-PEA_2_PbI_4_ /spiro-OMeTAD/Au	1.13	22.44	76.5	21.0	[75]
EDBEI	FTO/SnO_2_/perovskite /spiro-OMeTAD/Au	1.13	23.53	79.2	21.0	[76]
Poly(4-vinylpyridine)	ITO/SnO_2_/PbI_2_-MAI/PVP /spiro-OMeTAD/Au	1.15	21.74	80.9	20.2	[77]
Dimethyl itaconate	ITO/SnO_2_/FAI-MAI-MACl-DI /spiro-OMeTAD/Ag	1.15	24.90	80.8	23.0	[78]
PiP	FTO/PiP-SnO_2_/FA_0_._83_Cs_0_._17_ /i-BABr/spiro-OMeTAD/Au	1.17	24.85	83.0	24.1	[79]
EMIM DEP	ITO/SnO_2_/EMIM DEP /FAI-MAI-CsCl-PbI_2_ /spiro-OMeTAD/Au	1.17	24.19	81.9	23.2	[80]
BGCI	ITO/SnO_2_/BGCI/(FAPbI_3_)_x_(MAPbI_3_)_y_ /PEAI/spiro-OMeTAD/MoO_3_/Ag	1.19	25.00	82.7	24.4	[81]

## Data Availability

Data are available from the corresponding author upon reasonable request.
